# Shrub-mediated effects on soil nitrogen determines shrub-herbaceous interactions in drylands of the Tibetan Plateau

**DOI:** 10.3389/fpls.2023.1137365

**Published:** 2023-02-10

**Authors:** Guangshuai Cui, Francisco I. Pugnaire, Liu Yang, Wanglin Zhao, Rita Ale, Wei Shen, Tianxiang Luo, Eryuan Liang, Lin Zhang

**Affiliations:** ^1^ State Key Laboratory of Tibetan Plateau Earth System Science, Resources and Environment (TPESRE), Institute of Tibetan Plateau Research, Chinese Academy of Sciences, Beijing, China; ^2^ Estación Experimental de Zonas Áridas, Consejo Superior de Investigaciones Científicas, Almería, Spain; ^3^ University of Chinese Academy of Sciences, Beijing, China; ^4^ Institute of Science and Technology Information of Tibet Autonomous Region, Lhasa, China

**Keywords:** stress gradient hypothesis, water availability, soil nitrogen, arid shrub, facilitation

## Abstract

**Introduction:**

Shrub promotes the survival, growth and reproduction of understory species by buffering the environmental extremes and improving limited resources (i.e., facilitation effect) in arid and semiarid regions. However, the importance of soil water and nutrient availability on shrub facilitation, and its trend along a drought gradient have been relatively less addressed in water-limited systems.

**Methods:**

We investigated species richness, plant size, soil total nitrogen and dominant grass leaf δ^13^C within and outside the dominant leguminous cushion-like shrub *Caragana versicolor* along a water deficit gradient in drylands of Tibetan Plateau.

**Results:**

We found that *C. versicolor* increased grass species richness but had a negative effect on annual and perennial forbs. Along the water deficit gradient, plant interaction assessed by species richness (RII_species_) showed a unimodal pattern with shift from increase to decrease, while plant interaction assessed by plant size (RII_size_) did not vary significantly. The effect of *C. versicolor* on soil nitrogen, rather than water availability, determined its overall effect on understory species richness. Neither the effect of *C. versicolor* on soil nitrogen nor water availability affected plant size.

**Discussion:**

Our study suggests that the drying tendency in association with the recent warming trends observed in drylands of Tibetan Plateau, will likely hinder the facilitation effect of nurse leguminous shrub on understories if moisture availability crosses a critical minimum threshold.

## 1 Introduction

Positive and negative plant-plant interactions co-occur in plant communities (Callaway and Walker, 1997). The negative effect (i.e., competition) always happens between intra- or inter-species when they sharing limited living space or resources. The positive effect, facilitation, has strong impact in most harsh environments, where the mitigation of extreme climate by facilitator species can benefit many other species (Bertness and Callaway, 1994; Pugnaire et al., 1996; Brooker et al., 2008). Facilitation acts as a sort of insurance policy for plant communities in alpine regions (Cavieres et al., 2014; Cavieres et al., 2016; Pistón et al., 2016) and water-limited systems (Armas et al., 2011; Pugnaire et al., 2011; Parajuli et al., 2021; Pugnaire et al., 2021). Improving our knowledge of facilitation is, therefore, crucial to understand plant community dynamics and to predict plant community responses to a changing climate (Anthelme et al., 2014; Pugnaire et al., 2021).

Shrubs often act as nurse plants in arid habitats where water availability and soil nutrients are limiting factors (Butterfield et al., 2016), increasing species diversity (O'Brien et al., 2019) through buffering environmental conditions, increasing resource availability and/or protecting against herbivores (Maestre et al., 2009; Tirado et al., 2015; Zhang et al., 2018a). Shrubs could increase soil water availability through hydraulic lift (Richards and Caldwell, 1987; Prieto et al., 2011) or shading effect (Liu et al., 2021). However, shrubs can also have negative effects on soil water content mostly through interception loss in shrub canopies and enhanced utilization of soil water (Tielbörger and Kadmon, 2000; Darrouzet-Nardi et al., 2006; Hamerlynck et al., 2011). Nevertheless, in arid environments the effects of shrubs on soil moisture depends on the relationship between precipitation and differential evapo-transpiration rates under plants and in gaps (Butterfield et al., 2016). Formation of fertility islands underneath shrubs canopy represents another mechanism involved in the positive shrub-herbaceous interaction in drylands, mainly due to enhanced soil nutrient availability (Zhang et al., 2018b; Cai et al., 2020). In particular, leguminous shrubs have a significant effect on soil nitrogen accruement (Pugnaire et al., 1996; Zhang et al., 2011; Saixiyala et al., 2017). Accumulation of soil organic matter can also buffer soil water and thermal oscillations in the understory (Wezel et al., 2000; Maestre et al., 2001; Noumi et al., 2016). However, the relative contribution of shrub mediated effect on soil water and nutrient availability in the interaction between shrub and understory species have been less addressed in drylands.

Plant-plant interactions changes with resource availability and abiotic conditions. The stress gradient hypothesis (SGH) predicts an increase in the importance of facilitation with increasing environmental severity (Bertness and Callaway, 1994). But other models suggest that facilitation is not always the case, that would tend to be neutral interactions or even a switch from facilitation to competition with increasing severity in arid and semi-arid systems (Tielbörger and Kadmon, 2000; Maestre and Cortina, 2004; Michalet et al., 2006; Holmgren and Scheffer, 2010; O'Brien et al., 2017). Inconsistent plant interactions patterns along biotic gradient has been reported when different measurements (e.g., species richness, plant height, or reproduction) or statistical levels (e.g., paired vs. community scale) are involved (He et al., 2013; Soliveres et al., 2015; Liancourt et al., 2017). In addition, species turnover along gradients may blur predictions (Choler et al., 2001; Liancourt et al., 2005; Le Bagousse-Pinguet et al., 2012; Liancourt et al., 2017).

We addressed the differential effects of facilitation within and outside the canopy of a shrub, *Caragana versicolor*, along a water availability gradient in west drylands of Tibetan Plateau. West Tibet, with a mean elevation above 4,500 m a.s.l., is characteristic of a low rainfall, high radiation, and barren soil environment. As the dominant shrub species in the western Himalayan regions, *C. versicolor* is adapted to these habitats through its cushion canopy, deep roots, and N-fixing capacity (Kumar et al., 2016). In this study, we aimed to evaluate the relative effects of soil moisture and soil nutrients on shrub-understories interactions in a water-limited system. Considering that different functional groups show different adaptive strategies which may lead to inconsistent patterns of facilitator-understories interactions (Iyengar et al., 2017; Liancourt et al., 2017), we also compared the different effects of shrub facilitation on different functional groups. Our aims are to test: (1) whether the facilitation effects of *C. versicolor* on associated species increase with decreasing water availability; (2) whether soil nutrients play a stronger role than soil moisture in driving shrub-understory interactions in this arid region, and (3) whether grasses show stronger responses to facilitation than annual and perennial forbs because of their higher competitive ability. According to the SGH, we expected that facilitation intensity would increase as water availability decreases and that nutrients are more important than water in driving plant-plant interactions. Finally, we expected that shrubs have stronger facilitation on grasses than on other functional groups.

## 2 Materials and methods

### 2.1 Study area and sampling design

The study was conducted in Shigatse and Ngari areas in the Tibet Autonomous Region, China where the climate is cold and arid with a mean annual temperature ranging -8~2°C and annual precipitation of 50~500 mm which falls mainly in summer. The plant community is dominated by alpine shrubs (shrub-steppe) and alpine pastures (desert steppe), dominated by species such as *C. versicolor*, *Elymus nutans*, *Leymus* spp., *Stipa caucasica*, and *S. purpurea*. *C. versicolor* is the dominant shrub species, generally spanning 4250~5100 m in elevation. It is a Fabaceae deciduous species that usually grows in well-drained and loose sandy soils, with a 20 cm mean canopy height and a cover ranging 10%~50%.

**Figure 1:**
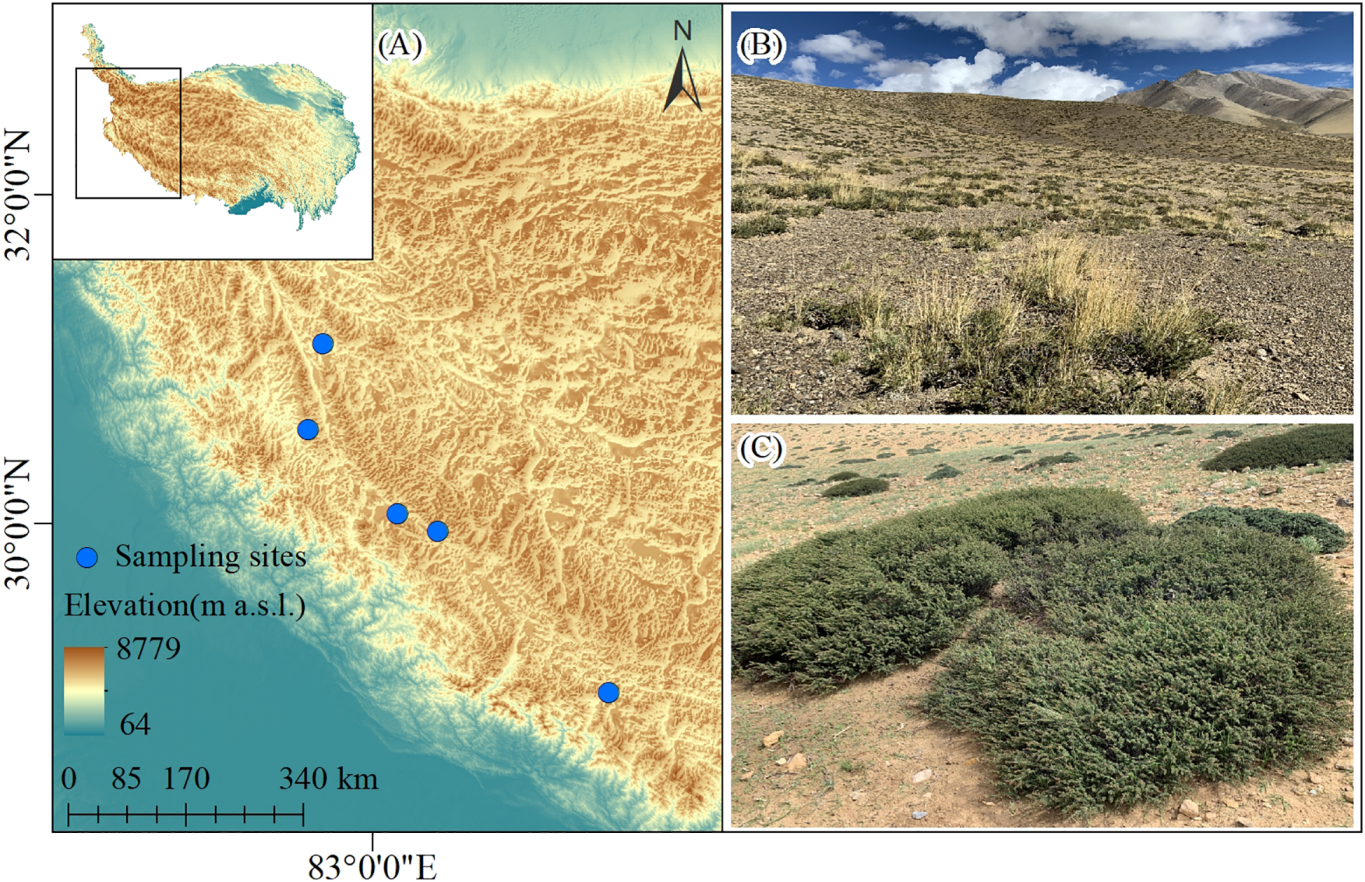
**(A)** A map showing the study area and sampling sites. Photos of the **(B)** *Caragana versicolor* community landscape and **(C)** shrub individuals in drylands of Tibetan Plateau.

In August 2019, we selected 5 sites along a longitudinal transect within a naturally-occurring water availability gradient. The locations and elevations of the sampling sites were measured by GPS (eTrex Venture, Garmin, USA) (**Figure 1**). At each site, we investigated 30 shrubs and 30 paired open areas equal in size to that of shrubs within an area of approximately 1 ha. Shrub understory area was calculated as the area of an ellipse by measuring the largest diameter and its perpendicular. The shape of every shrub was simulated with wire, and the wire was randomly placed 1 meter near the shrub edge to investigate the paired open area. All vascular plants growing within the paired plots were identified, and the mean size of each species was measured. For the erect plants, which size were measured as individual height, the length of stem was measured as plant size instead for the prostrate species. A total of 150 paired plots were explored using the protocol described above.

In each site, the first 5 paired plots were selected for sampling soil (0~20 cm) and the same dominant grass leaves under *C. versicolor* and in the paired open area (**Supplementary Table 1**). Soil total nitrogen content was determined using an elemental analyzer (2400 II CHNS; Perkin Elmer, Boston, MA, USA). We failed to measure soil moisture across the whole water availability gradient, only one site located in the westernmost gained the soil moisture. Volumetric soil moisture at -10 cm underneath the shrub canopy and in the open area were measured with HOBO weather stations (Onset Inc., Bourne, MA, USA), which were recorded since Aguste 8th 2015 to Journey 1st 2020 with 1 hour interval, the missing data is due to equipment failure (**Supplementary Figure 1**). In C_3_ plants, stable carbon isotope composition in leaves (δ^13^C) can provide an integrated measurement of internal-physiological and external-environmental conditions during the growing season (Farquhar et al., 1989; Dawson et al., 2002). In dry habitats, leaf δ^13^C is highly sensitive to water availability, and Ale et al. (2018) found that widespread species leaf δ^13^C would be used as an indicator of water availability in dry mountainous regions like the Himalayas. In this study, leaf δ^13^C of dominant grass species occurring within and outside the shrub canopy was used to characterize soil water availability. The δ^13^C ratio in grasses leaves, relative to a Pee Dee Belemnite (PDB) standard, was determined with an elemental analyzer coupled to a stable isotope mass spectrometer (Flash EA + Delta V, Thermo Fisher Scientific Inc., Waltham, MA, USA) at the State Key Laboratory of Tibetan Plateau Earth System Science, Resources and Environment (TPESRE), Chinese Academy of Sciences. The overall precision of the δ^13^C analysis was 0.1‰.

### 2.2 Data analyses

The intensity of interactions between *C. versicolor* and associated species was assessed by species richness and plant size using the relative interaction index, RII_species_ and RII_size,_ respectively (Armas et al., 2004) as $\text{RII}=\frac{\text{Xshrub}-\text{Xopen}}{\text{Xshrub}+\text{Xopen}}$ , where X is either species richness or plant size under the shrub (X_shrub_) or in its paired open area (X_open_). We grouped plant species as grasses, annual forbs, and perennial forbs. RII was calculated at community and functional group levels, respectively.

In order to assess whether our selection of 30 plots in each site is good enough to catch the regional species pool, the rarefaction analysis was run for each site (**Supplementary Figure 2**). We constructed species accumulation curves to quantify species richness under *C. versicolor* shrubs (S_shrub_), open areas (S_open_), and at the community level (S_total_) in each site (Badano et al., 2006). To estimate S_total_ per site, we generated datasets combining data taken under *C. versicolor* and in open areas. For each rarefaction curve, 500 resamples were randomly drawn without replacement for each sample size (from one sample to the maximum number of samples). In such analyses, the point along the sampling effort axis where the species accumulation curve reaches an asymptote indicates the number of samples needed to successfully sample the full assemblage of species (Gotelli and Colwell, 2001). All rarefaction analyses were carried out using EstimateS 9.1 software (Colwell et al., 2012). To assess the magnitude of the increase in species richness at the community level due to the presence of *C. versicolor*, we calculated the proportion of increase in herbaceous species richness as $\text{ISR}=\frac{\text{Stotal}-\text{Sopen}}{\text{Stotal}}$ (Cavieres et al., 2016). This index provides a qualitative assessment of the magnitude of the effect of shrubs on species richness at the scale of the entire local community.

To assess the difference in species compositions along the gradient, we performed a Principal Coordinate Analysis (PCoA) on a Bray-Curtis dissimilarity matrix. This analysis was performed for the quadrats located away from the shrub only (open areas). The significance of differences between sites was tested by ANOSIM with 9999 permutations. PCoA and screening were performed using the R version 4.1.2 with ape package (https://cran.r-project.org/web/packages/ape/index.html) and vegan package (https://cran.r-project.org/web/packages/vegan/index.html).

There is a scarcity of weather stations in the study area, so that, to obtain comparable long-term climate data, monthly mean temperature and precipitation data were obtained from the WorldClim 2.1 dataset with a spatial resolution of 1 km^2^ (https://worldclim.org/data/worldclim21.html) (Hijmans et al., 2005) using the geographical coordinates of the sampling sites. Temperature data were corrected by a lapse rate of 6°C km^-1^ (Kattel et al., 2015). WorldClim data have been widely used for calculating moisture indices to determine habitat severity in different mountain systems where long-term climate records are rarely available. Based on monthly WorldClim data, the annual Thornthwaite water deficit (WD) was calculated for each sampling site as follows: WD = P - PET, where P is the mean annual precipitation and PET is the mean annual potential evapotranspiration (Thornthwaite and Mather, 1955).

We used a one-sample t-test to assess whether RII values differed significantly from 0. Site differences in RII were tested by one-way analysis of variance followed by *post hoc* Tukey/Duncan test or Dunnett’s T3 test (for homogeneity of variances). The effects of shrub on micro-environment (soil nitrogen and soil water availability) and understory species (species richness and plant size) were assessed by the difference in understory canopy (minuend) and in the open area (subtrahend). General linear regressions of the effects of shrub on understory plants against the effects of shrub on micro-environment were performed at the plot level. All statistical analyses were performed using SPSS 18.0 for Windows, and all significant differences were at *P* < 0.05. The sampling map was drawn by ArcGIS version 10.8 for Desktop (Environmental Systems Research Institute, Inc.). The elevation generated for the map was downloaded from WorldClim 2.1 with 30s resolution (https://worldclim.org/data/worldclim21.html).

## 3 Results

### 3.1 Species composition along the water availability gradient

**Figure 2:**
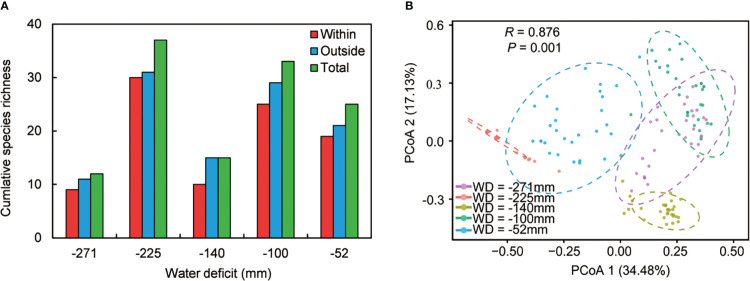
**(A)** Species richness under *Caragana versicolor* (within), in open areas (outside) and at the community level (total) along the water deficit gradient in drylands of Tibetan Plateau. **(B)** Ordination diagram showing Principal Coordinates Analysis (PCoA) performed for species composition in open areas.

We recorded 68 associated species in our five sampling sites across the water availability gradient (**Supplementary Table 2**). Species richness reflected nonlinear patterns in different microhabitats, including under *C. versicolor*, open areas, and at the community level (**Figure 2A**). Species richness in open areas was higher than under *C. versicolor* at all sites, and species richness in the whole community (i.e., understory and open areas combined) was higher than under *C. versicolor* and open areas (**Figure 2A**). Principal Coordinate Analysis (PCoA) indicated that the species composition in each of our five sampling sites was significantly different from the others (**Figure 2B**, *R* = 0.876, *P* = 0.001).

We found positive ISR values across sites except at site with WD = -100 mm, indicating that, overall, shrubs enhance species richness at the community level (**Table 1**). Some specific species were recorded either under *C. versicolor* or in open areas across the water availability gradient (**Table 1**), suggesting that some species had strong habitat preferences.

**Table 1:** Water deficit (WD), the proportion of increase in herbaceous species richness (ISR) and species occurrence under *Caragana versicolor* or in open areas across 5 sampling sites in drylands of Tibetan Plateau.

WD/mm	ISR	Species only occur under *C. versicolor*	Species only occur in open area
-271	0.083	*Krascheninnikovia compacta*	*Artemisia desertorum*; *Euphorbia tibetica*
-225	0.175	*Allium* spp.; *Ephedra* spp.; *Onosma confertum*; *Sedum* spp.; *Silene moorcroftiana*; *Silene aprica*	*Astragalus arnoldii*; *Androsace graminifolia*; *Carex* spp.; *Phyllolobium tribulifolium*; *Potentilla bifurca*; *Stellera chamaejasme*
-140	0	NA	*Alyssum canescens*; *Artemisia stracheyi*; *Lasiocaryum densiflorum*; *Oxytropis microphylla*;*Oxytropis* spp.
-100	0.111	*Artemisia vexans*; *Saussurea* spp.; *Silene aprica*; *Urtica* spp.	*Artemisia desertorum*; *Artemisia wellbyi*; *Fabaceae* spp.; *Kobresia* spp.; *Pedicularis alaschanica*; *Potentilla* spp.; *Salsola monoptera*; *Swertia hispidicalyx*
-52	0.154	*Brassicaceae* spp.; *Dysphania schraderiana*; *Poaceae* spp.; *Silene aprica*	*Artemisia* spp.; *Carex* spp.; *Chamaerhodos sabulosa*; *Oxytropis microphylla*; *Phyllolobium heydei*; *Poaceae* spp.

### 3.2 Effects of *C. versicolor* on species richness and plant size of its understory community

**Figure 3:**
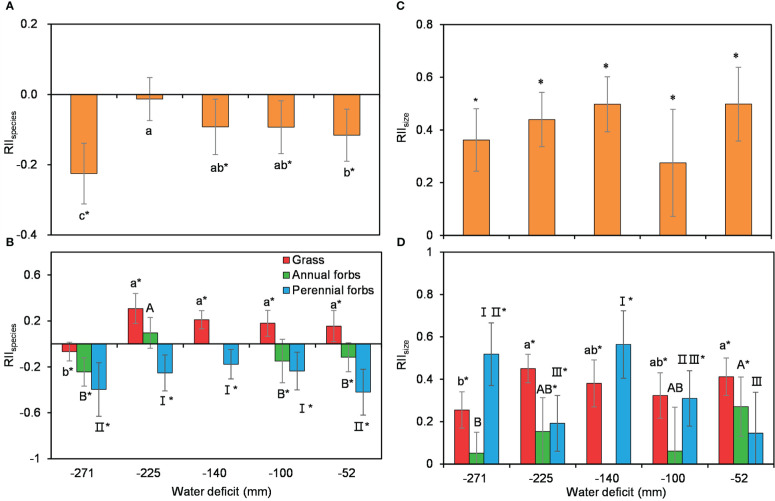
Variations in the interaction effects between *Caragana versicolor* and understory species assessed by species richness RII_species_ **(A, B)** and plant size RII_size_ **(C, D)** for total associated species (a and c) and across different functional groups (b and d) along the water deficit gradient in drylands of Tibetan Plateau. Different letters and roman numbers indicate significant differences in RII_species_ or RII_size_ between sites; asterisks show that RII_species_ or RII_size_ is significantly different from 0.

Shrub size, was assessed by the product of the long and minor axes of shrub canopy, as a soft index of shrub age, had no significant effect on understory species richness nor plant size (**Supplementary Figure 3**). Using all species combined, RII_species_ was mostly negative across the water availability gradient, showing a unimodal trend that suggested a rather strong competition between *C. versicolor* and its associated understory community (**Figure 3A**). For the different functional groups, RII_species_ showed different patterns. RII_species_ for grasses was significantly positive except at the driest site (**Figure 3B**). By contrast, RII_species_ for annual and perennial forbs was generally negative, suggesting competition between shrubs and these two functional groups (**Figure 3B**). Similarly, RII_species_ including all functional groups also showed a unimodal, negative trend along the water availability gradient (**Figure 3B**).

The effects of *C. versicolor* on forbs growth, measured as RII_size_, were significantly positive for all species combined and species in different functional groups, indicating a positive effect on plant growth (**Figures 3C, D**). RII_size_ for all species combined showed a similar trend, with no significant changes along the water availability gradient (**Figures 3C, D**).

### 3.3 Variations in soil total nitrogen and grass leaf δ^13^C and their effects on RII_species_ and RII_size_

**Figure 4:**
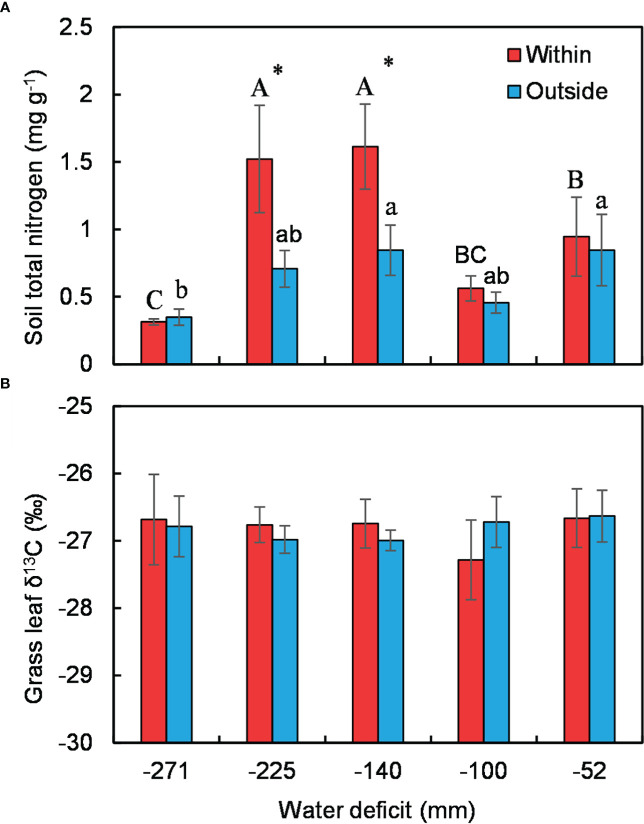
Variations in soil total nitrogen **(A)** and grass leaf δ^13^C **(B)** under *Caragana versicolor* and in open areas along the water deficit gradient in drylands of Tibetan Plateau. Different letters indicate significant differences in soil total N or δ^13^C between sites; asterisks show significant differences between the shrub understory and open areas.

Soil nitrogen was generally higher under *C. versicolor* than in open areas except at the driest site (**Figure 4A**). Soil nitrogen in the understory showed a similar unimodal trend along the water deficit gradient, while there was no significant trend in open areas (**Figure 4A**). Shrub size had nothing to do with the mediated effect of shrubs on soil total nitrogen (**Supplementary Figure 4**). There were no differences in grass leaf δ^13^C across the water gradient, neither within nor outside *C. versicolor* canopies (**Figure 4B**).

**Figure 5:**
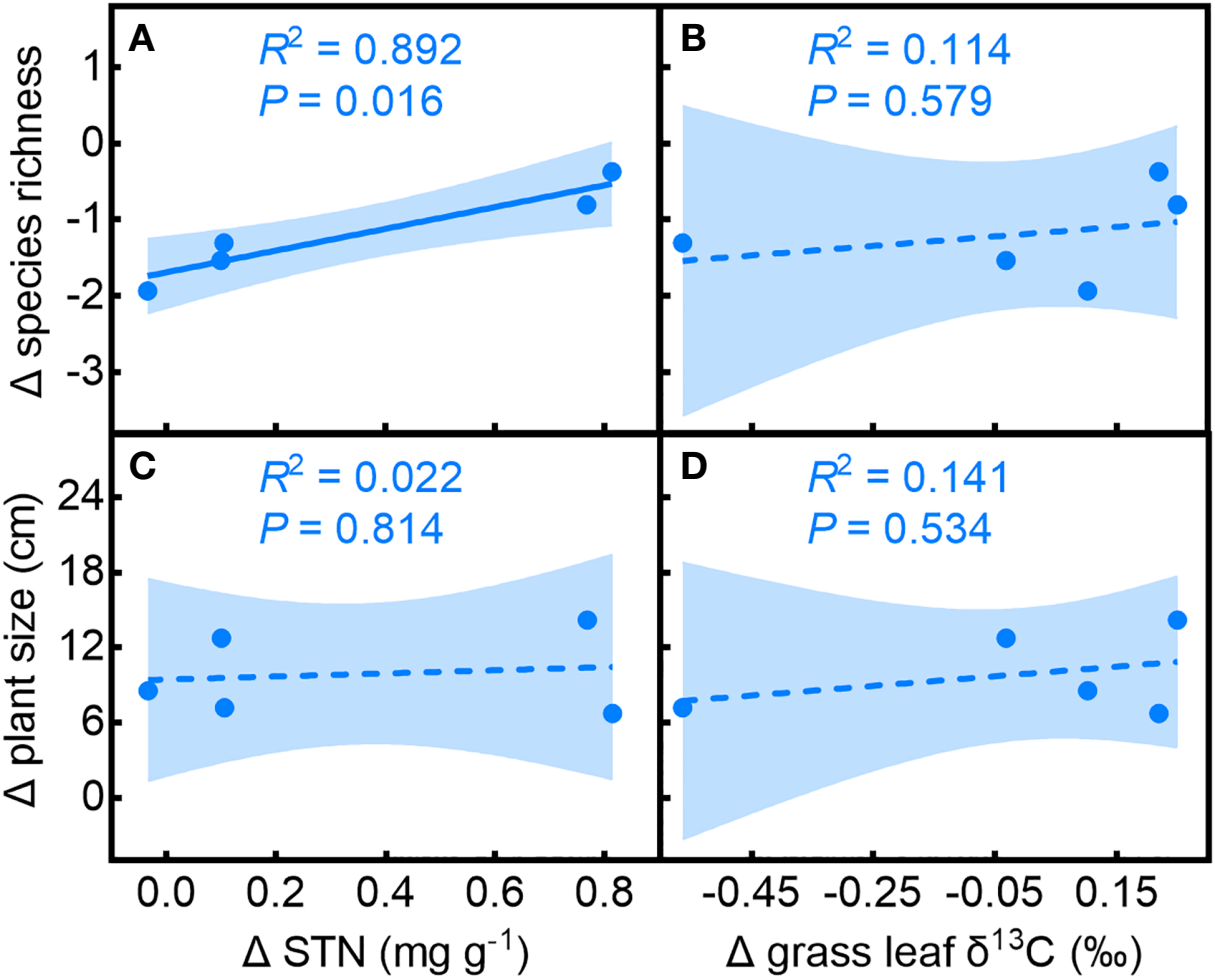
Relationships of the effects of *Caragana versicolor* on **(A, B)** species richness (Δ species richness) and **(C, D)** plant size (Δ plant size) to the effect on soil N (Δ soil N) and grass leaf δ^13^C (Δ grass leaf δ^13^C) in drylands of Tibetan Plateau.

The effect of the shrub on species richness compared to open spaces (Δ species richness) was related to its effects on soil N (Δ soil nitrogen; **Figure 5A**, *R^2^* = 0.892, *P* = 0.016) and had no significant relationship with Δ grass leaf δ^13^C (**Figure 5B**, *R^2^* = 0.114, *P* = 0.579). The effect of shrubs on understory plant size (Δ plant size) did not show significant relationships with Δ soil nitrogen (**Figure 5C**, *R^2^* = 0.022, *P* = 0.814) or Δ grass leaf δ^13^C (**Figure 5D**, *R^2^* = 0.141, *P* = 0.533), suggesting that the effect of *C. versicolor* on soil nitrogen, rather than water availability, determines its overall effect on understory species richness. Neither the effect of *C. versicolor* on soil nitrogen nor water availability affected plant size.

## 4 Discussion

We recorded inconsistent patterns of plant interaction assessed by species richness and plant size with decreasing water availability in drylands of Tibetan Plateau (**Figures 3A, C**), the unimodal trend of the effect of *C. versicolor* on species richness departure from the prediction of the SGH (Bertness and Callaway, 1994), but was consistent with reports in other dry systems (Liancourt et al., 2017; O'Brien et al., 2017; Zhang et al., 2018a). We found that *C. versicolor* significantly increased understory soil nitrogen but had a negligible effect on soil water availability (**Figures 4A, B**). The effect of *C. versicolor* on understory species richness was positively related to its effect on soil nitrogen (**Figure 5**), suggesting that the effect of shrub on soil N, rather than on soil moisture, determines the positive effect of *C. versicolor* on species richness. As we expected, grass gained more facilitation from *C. versicolor* than annual and perennial forbs (**Figure 3A**), likely due to its higher competitive capacity for limited resources compared with other functional groups.

### 4.1 Effects of arid shrub on associated understory community

Nurse plants have positive effects on the germination, survival, and growth of other species under their canopies in harsh environments such as alpine and arid habitats (Brooker et al., 2008; Michalet and Pugnaire, 2016; Liancourt and Dolezal, 2021). However, for *C. versicolor* in drylands of Tibetan Plateau, we found opposite trends regarding species richness, which were lower under the shrub canopy. The lower species richness under the canopy than in open spaces between shrubs has been reported also by Liancourt et al. (2017) in the same species in arid Trans-Himalayas but contrasts with reports of positive effects of shrubs on understory species richness in high mountains elsewhere (Ballantyne and Pickering, 2015; Pistón et al., 2016; Iyengar et al., 2017; Parajuli et al., 2021), being most likely an effect of species-specific traits (Callaway, 2007).

We found soil moisture underneath the shrub canopy was generally lower than that in the open area (**Supplementary Figure 1**), also the water availability indicator, leaf δ^13^C of grasses, was slightly higher within *C. versicolor* canopies than outside at most study sites across the water gradient, suggesting that shrubs do not increase soil moisture as do in other facilitator species (Prieto et al., 2011; Tirado et al., 2015; Liu et al., 2021). Water availability is the most important factor limiting plant survival in dry regions, where increasing water availability translates into increased plant survival (Liancourt et al., 2005). In fact, the negative effect of *C. versicolor* on its understory species is most likely a consequence of competition for soil water. In addition, the negative effect of *C. versicolor* on the plant survival of associated species would related to its canopy architecture, which branches are dense, forming a tight, hemispheric canopy that provides shade to understory species in this dry environment. Similar results were also reported by Al Hayek et al. (2015) for the spiny cushion shrub, *Onobrychis cornuta*, which showed strong facilitation in phenotypes with loose canopy and no significant or negative effects in phenotypes with tight canopy. A similar effect was reported for *Cytisus galianoi* in the Sierra Nevada mountains in Spain (Pistón et al., 2018).

We found that *C. versicolor* contributed to increasing community-scale species richness at most sites (ISR > 0), since some species were only found under shrub canopies while others preferred open habitats. For instance, *Allium* spp., *Onosma confertum*, *Silene aprica*, and *S. moorcroftiana* are tall enough to overtop the *C. versicolor* canopy and therefore are not completely shaded by the shrub, taking advantage of soil nutrients improvement by the shrub. On the contrary, some small Cyperaceae species such as *Carex* spp. and *Kobresia* spp., and Fabaceae species with symbiotic nitrogen fixation such as *Astragalus arnoldii*, *Oxytropis microphylla*, *Phyllolobium heydei* and *Phyllolobium tribulifolium*, only occur in open areas, suggesting they are excluded from the understory by shading. Similarly, some light-demanding species such as *Artemisia desertorum*, *A. stracheyi*, and *A. wellbyi*, which are also unpalatable, were found only in open areas, most likely because they do not stand competition by the shrub (Haase et al., 1996). These cases illustrate how the microenvironmental heterogeneity induced by shrubs affects patterns of species distribution in the community (Armas et al., 2011; Ballantyne and Pickering, 2015; Madrigal-González et al., 2016; Pistón et al., 2016).

In contrast to species richness, we found that RII_size_ was generally positive, indicating that *C. versicolor* increases plant size of associated species. The effect of *C. versicolor* on plant size may be a consequence of positive shading effect by the shrub. Shrub canopies reduce radiation and wind speed (Padilla and Pugnaire, 2009; Holmgren et al., 2012; Pistón et al., 2016), improving survival of understory species by preventing desiccation. This mechanism should become important in a high-elevation environments such as the Tibetan Plateau, with high radiation and strong winds, where low CO_2_ levels increase stomatal conductance and pose high risk of desiccation. Although shading may lead to taller plants through etiolation, the benefits of living in the understory may exceed the drawbacks of shade in this otherwise well-lit environment. Additionally, the facilitative effect of *C. versicolor* on plant size is likely related to the protection against livestock grazing, as plants growing under shrub patches are less accessible to herbivores, therefore experiencing lower grazing pressure (Tirado et al., 2015; Xie et al., 2017; Parajuli et al., 2021).

Considering different functional groups, we found that *C. versicolor* increased grass species richness but had a negative effect on forbs. The higher competitive ability of grasses compared to other functional groups has been shown in several experiments (Maestre and Cortina, 2004; Noumi et al., 2015; Liu et al., 2021) due to its higher root investment and resources uptake abilities (Gómez-Aparicio, 2009; Gross et al., 2009; Michalet et al., 2015). In addition, exclusion of forbs by *C. versicolor* could be also due to the generally high N demand of forbs, resulting in strong competition between forbs and shrub for N.

### 4.2 Shrub facilitation along a water availability gradient

The net balance of benefactor-beneficiary species interaction is species-specific and depends on resource availability (Soliveres et al., 2015; Michalet and Pugnaire, 2016). The SGH predicts a higher frequency of facilitative interactions as resource limitation increases, since facilitator species buffer severe environmental conditions (Bertness and Callaway, 1994). We found that the interaction between *C. versicolor* and associated species, assessed by species richness and plant size (RII_species_ and RII_size_, respectively), showed different trends along the water deficit gradient. RII_species_ showed a unimodal trend with decreasing water availability, while RII_size_ did not change significantly. The effect of *C. versicolor* on understory species richness (Δ species richness) was positively related to its effect on soil nitrogen (Δ soil nitrogen), but had no significant relationship with Δ leaf δ^13^C in grasses, indicating that the effect of shrub on soil N, rather than on soil moisture, determines the positive effect of *C. versicolor* on species richness. Neither the effect of *C. versicolor* on soil nitrogen nor on water availability affected plant size. This result highlighted the importance of the interaction mechanism to accurately predict the intensity and direction of facilitator-beneficiaries in water-limited systems.

The shift from facilitation to competition under drought conditions may occur when the facilitator species could not take up enough resources for its own subsistence (Maestre and Cortina, 2004; O'Brien et al., 2017; Zhang et al., 2018a). Several studies found that the unimodal trends of plant-plant interactions in water-limited systems were associated with the inability of the facilitator to provide resources (usually, soil water) at the very end of a stress gradient (Michalet et al., 2006; Michalet, 2007; Michalet et al., 2014; Zhang et al., 2016; O'Brien et al., 2017). However, we found that the decrease of *C. versicolor* on facilitation on species richness at the extremely dry site was related to the decreasing effect on soil nitrogen. Previous studies have proved that soil-mediated effect on soil play an important role on shrub-understories interactions in drylands. For instance, a manipulative experiment found that the roots of *Dasiphora fruticosa* had stronger effect on understory species composition than the shrub canopy in eastern Tibetan Plateau (Wang et al., 2017). Similar results have also been shown by other studies, which found that understory soil had positive effect on associated species, while shrub canopy had competitive or neutral effects in arid Mediterranean systems (Noumi et al., 2016; Lozano et al., 2020; Chaieb et al., 2021). Soil contribution to facilitation is associated with increased nutrient content in shrub patches or indirectly mediated by soil microorganisms (Rodríguez-Echeverría et al., 2016; Pugnaire et al., 2019). Above mechanism is particular important for leguminous nurse shrub, because their symbiotic N fixation is highly sensitive to water availability. Nitrogenase activities rely on the amount of carbohydrates supplied to nodules, and the supply decreases under water-limited conditions (Marino et al., 2007; Arfin Khan et al., 2014).

The functional strategy (i.e., stress tolerance and competitive ability) of the species involved in the interactions may also affect plant interactions (Michalet et al., 2014). Understory species along the water availability gradient were not always the same and environmental stress should select species that are more drought-tolerant. The turnover of beneficiary species with increasing stress often determines the plant interactions along stress gradients (Choler et al., 2001; Liancourt et al., 2005; Le Bagousse-Pinguet et al., 2012; Liancourt et al., 2017). When both interacting species are stress-tolerant, facilitation can be expected at moderate stress while competition may occur at high-stress levels (Maestre and Cortina, 2004). This may lead to a collapse of facilitation at the extreme end of the stress gradient (Maestre et al, 2009). Our data show that understory species composition in each sampling site significantly differed from other sites. Species are replaced by more drought-tolerant species at the driest site, where they are less likely to obtain a net benefit from the nurse shrub.

Multiple evidences indicate that recent climate change exerts large impacts on the structure and dynamics of plant terrestrial ecosystems, especially in hash systems such as alpine and arid regions (Peñuelas et al., 2013; Piao et al., 2014). In high elevation environments of the Himalaya mountains, climate warming in recent decades along with lower precipitations has led to an increase in water deficit (Yao et al., 2012; Sigdel et al., 2018). Our study found that the facilitation of dominant nurse shrub on soil nitrogen and understory species richness decreased at the driest site in drylands of the Tibetan Plateau, suggesting that the facilitation effect likely decrease if warming-induced water availability crosses a critical minimum threshold. Indeed, the negative effect of climate warming on plant growth and community dynamic has been evidenced, showing that recent warming caused soil moisture deficit and exceeded the thermal optimum of shrub recruitment in high-elevation and high-latitude regions (Francon et al., 2021; Sigdel et al., 2021; Wang et al., 2021; Lu et al., 2022).

## 5 Conclusion

The dominant leguminous shrub *C. versicolor* in drylands of the Tibetan Plateau had positive effect on grass species richness, but shown a negative impact on species richness of annual and perennial forbs. Also, *C. versicolor* exerted a positive effect on plant size of associated species that did not change along the water deficit gradient. The effect of *C. versicolor* on species richness showed a unimodal trend with decreasing water availability, closely related to the decreasing amelioration of soil nitrogen under extreme dry conditions. Our results suggest that the tendency of drought associated with warming in drylands on the Tibetan Plateau will likely hinder the facilitation effect of nurse leguminous shrub on understories if moisture availability crosses a critical minimum threshold.

## Data availability statement

The original contributions presented in the study are included in the article/**Supplementary Material**. Further inquiries can be directed to the corresponding author.

## Author contributions

LZ and EL designed the study; LZ, GC, and LY collected and measured the samples. All authors contributed to data analyses, interpretation and writing. All authors contributed to the article and approved the submitted version.
