# Supplementary material for: Shrub-mediated effects on soil nitrogen determines shrub-herbaceous interactions in drylands of the Tibetan Plateau
Source: Front Plant Sci. 2023 Feb 10;14:1137365. doi: 10.3389/fpls.2023.1137365 (PMC9950575; doi:10.3389/fpls.2023.1137365)
Supplement: Supplementary file 1 [file DataSheet_1.docx]

**Supplementary Table 1.** Mean annual precipitation (MAP), mean annual temperature (MAT), water deficit (WD) and collected dominant grass species under *Caragana versicolor* and in open areas across 5 sampling sites in drylands of Tibetan Plateau.

| Sampling sites | MAP/mm | MAT/°C | WD/mm | Dominant grass species |
| --- | --- | --- | --- | --- |
| 1 | 81 | -2.68 | -271 | *Stipa roborowskyi* |
| 2 | 312 | -0.03 | -225 | *Stipa breviflora* |
| 3 | 274 | -4.60 | -140 | *Stipa roborowskyi* |
| 4 | 392 | -1.71 | -100 | *Stipa roborowskyi* |
| 5 | 396 | -3.62 | -52 | *Stipa purpurea* |

**Supplementary Table 2.** Species list recorded at 5 sampling sites in drylands of Tibetan Plateau.

| NO. | Species | Family | NO. | Species | Family |
| --- | --- | --- | --- | --- | --- |
| 1 | *Allium* spp | *Amaryllidaceae* | 35 | *Incarvillea younghusbandii* | *Bignoniaceae* |
| 2 | *Anaphalis xylorhiza* | *Asteraceae* | 36 | *Kali monopterum* | *Amaranthaceae* |
| 3 | *Androsace graminifolia* | *Primulaceae* | 37 | *Krascheninnikovia compacta* | *Amaranthaceae* |
| 4 | *Artemisia* spp1 | *Asteraceae* | 38 | *Lasiocaryum densiflorum* | *Boraginaceae* |
| 5 | *Artemisia* spp2 | *Asteraceae* | 39 | *Lepidium capitatum* | *Brassicaceae* |
| 6 | *Artemisia demissa* | *Asteraceae* | 40 | *Leymus* spp | *Poaceae* |
| 7 | *Artemisia desertorum var. tongolensis* | *Asteraceae* | 41 | *Morina kokonorica* | *Caprifoliaceae* |
| 8 | *Artemisia nanschanica* | *Asteraceae* | 42 | *Onosma confertum* | *Boraginaceae* |
| 9 | *Artemisia stracheyi* | *Asteraceae* | 43 | *Oxytropis* spp1 | *Fabaceae* |
| 10 | *Artemisia vexans* | *Asteraceae* | 44 | *Oxytropis* spp2 | *Fabaceae* |
| 11 | *Artemisia wellbyi* | *Asteraceae* | 45 | *Oxytropis microphylla* | *Fabaceae* |
| 12 | *Aster gouldii* | *Asteraceae* | 46 | *Oxytropis proboscidea* | *Fabaceae* |
| 13 | *Aster semiprostratus* | *Asteraceae* | 47 | *Pedicularis alaschanica* | *Orobanchaceae* |
| 14 | *Astragalus* spp | *Fabaceae* | 48 | *Phyllolobium heydei* | *Fabaceae* |
| 15 | *Astragalus arnoldii* | *Fabaceae* | 49 | *Phyllolobium tribulifolium* | *Fabaceae* |
| 16 | *Brassicaceae* spp1 | *Brassicaceae* | 50 | *Poa* spp | *Poaceae* |
| 17 | *Brassicaceae* spp1 | *Brassicaceae* | 51 | *Poaceae* spp1 | *Poaceae* |
| 18 | *Carex* spp1 | *Cyperaceae* | 52 | *Poaceae*spp2 | *Poaceae* |
| 19 | *Carex* spp2 | *Cyperaceae* | 53 | *Potentilla* spp | *Rosaceae* |
| 20 | *Carex* spp3 | *Cyperaceae* | 54 | *Potentilla bifurca var. humilior* | *Rosaceae* |
| 21 | *Carex sargentiana* | *Cyperaceae* | 55 | *Rhodiola* spp | *Crassulaceae* |
| 22 | *Chamaerhodos sabulosa* | *Rosaceae* | 56 | *Saussurea*spp | *Asteraceae* |
| 23 | *Corispermum tibeticum* | *Amaranthaceae* | 57 | *Silene moorcroftiana* | *Caryophyllaceae* |
| 24 | *Corydalis*spp | *Papaveraceae* | 58 | *Silene aprica* | *Caryophyllaceae* |
| 25 | *Delphinium* spp | *Ranunculaceae* | 59 | *Stellera* spp | *Thymelaeaceae* |
| 26 | *Deyeuxia* spp | *Poaceae* | 60 | *Stevenia canescens* | *Brassicaceae* |
| 27 | *Dontostemon glandulosus* | *Brassicaceae* | 61 | *Stipa breviflora* | *Poaceae* |
| 28 | *Dracocephalum heterophyllum* | *Lamiaceae* | 62 | *Stipa purpurea* | *Poaceae* |
| 29 | *Dysphania schraderiana* | *Amaranthaceae* | 63 | *Stipa roborowskyi* | *Poaceae* |
| 30 | *Elymus nutans* | *Poaceae* | 64 | *Swertia hispidicalyx* | *Gentianaceae* |
| 31 | *Ephedra* spp | *Ephedraceae* | 65 | *Thalictrum* spp | *Ranunculaceae* |
| 32 | *Eremogone bryophylla* | *Caryophyllaceae* | 66 | *Urtica* spp | *Urticaceae* |
| 33 | *Euphorbia tibetica* | *Euphorbiaceae* | 67 | *Viola* spp | *Violaceae* |
| 34 | *Hypecoum leptocarpum* | *Papaveraceae* | 68 | *Youngia* spp | *Asteraceae* |

**
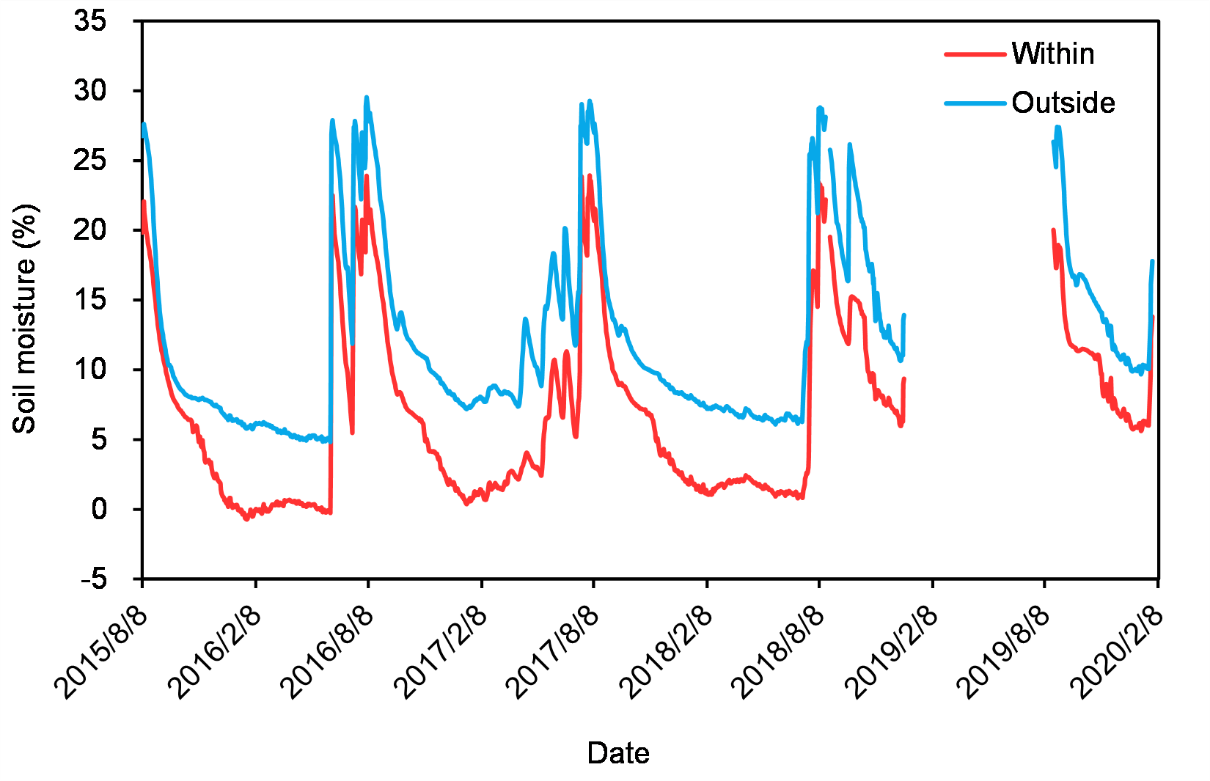
**

**Supplementary Figure 1.** Variations of soil moisture under *Caragana versicolor* canopy (within) and in open areas (outside) at the sampling site with water deficit -271 mm during Aguste 8th 2015 to Journey 1st 2020.

**
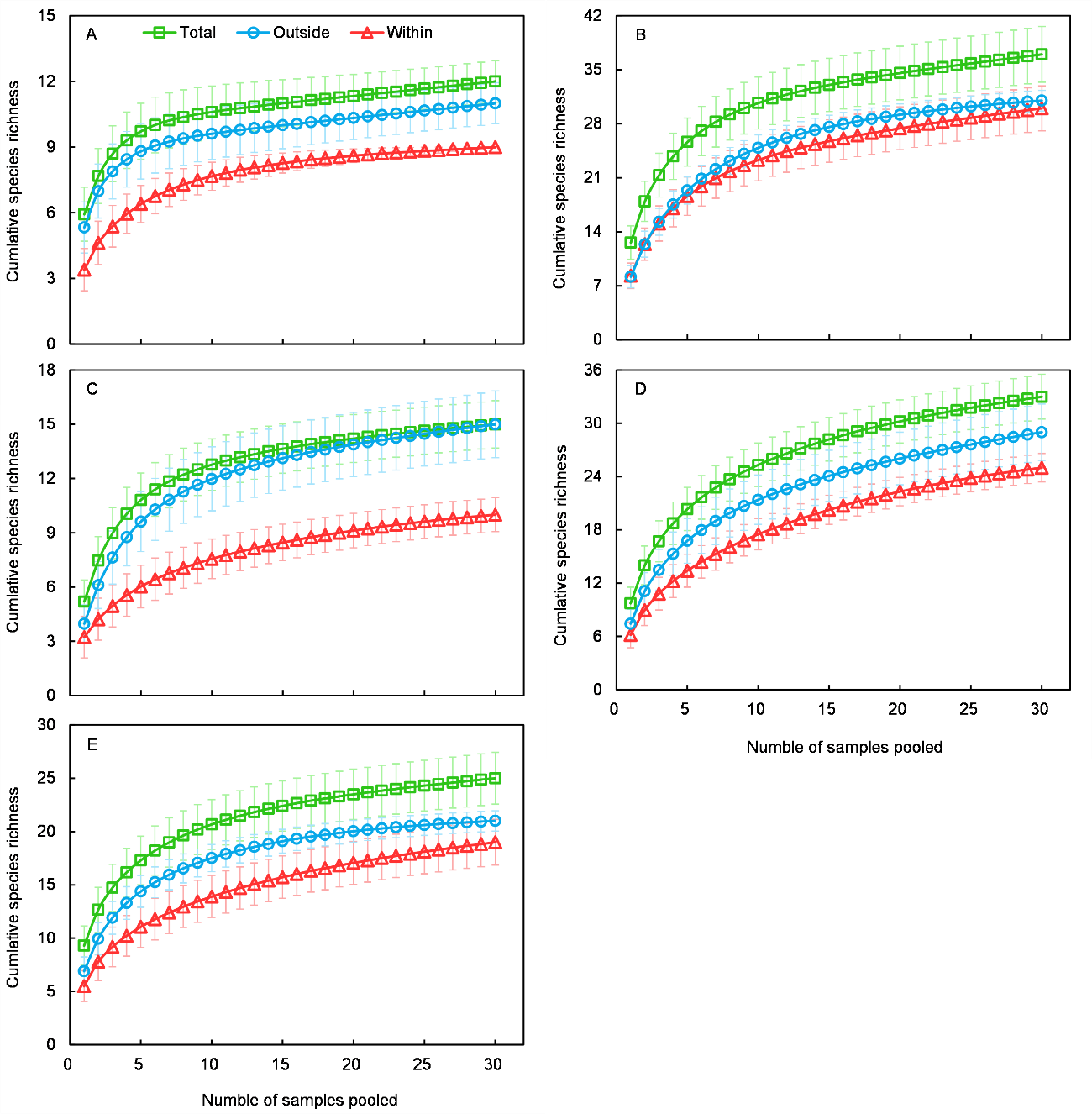
**

**Supplementary Figure 2.** Estimated values of species richness for different habitats, including community-level (total), open area (outside) and under *Caragana versicolor* (within) across the water deficit gradient in drylands of Tibetan Plateau. (A) WD = -271 mm, (B) WD = -225 mm, (C) WD = -140 mm, (D) WD = -100 mm and (E) WD = -52 mm.


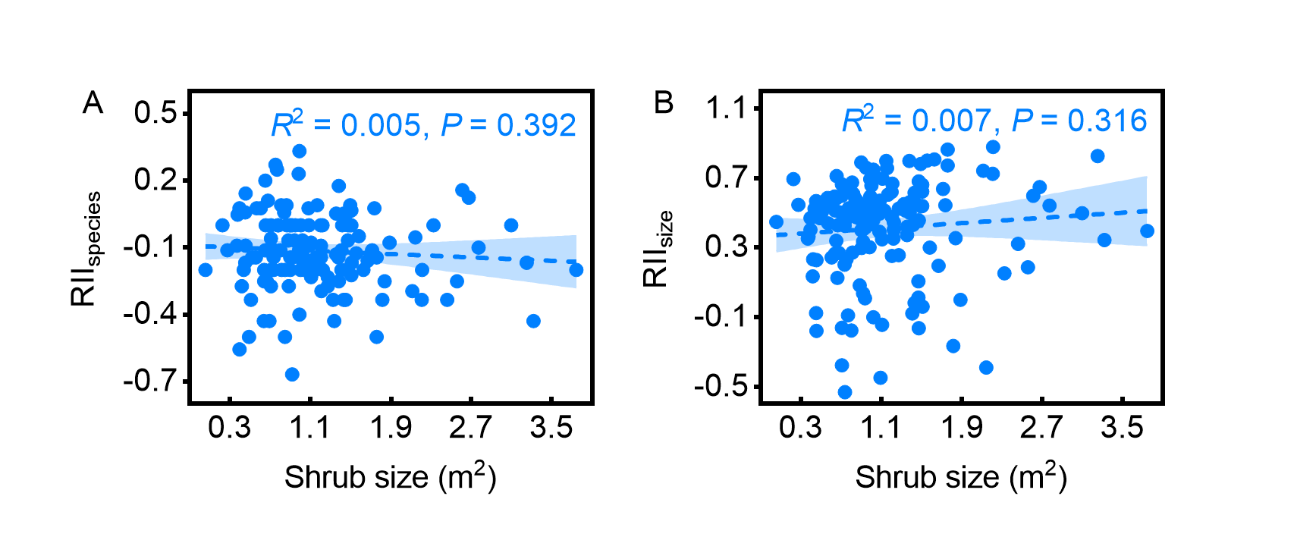


**Supplementary Figure 3.** Relationships of the interactions between *Caragana versicolor* and associated plant species assessed by (A) species richness (RII_species_) and (B) plant size (RII_size_) on its canopy size across 5 sampling sites in drylands of Tibetan Plateau.


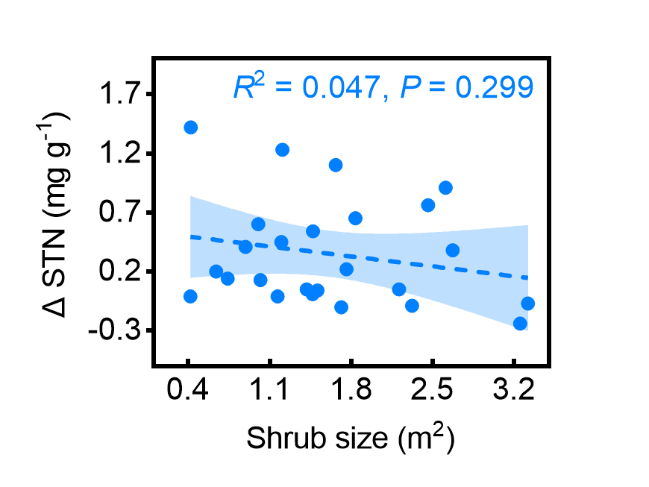


**Supplementary Figure 4.** Relationship of the effects of *Caragana versicolor* on soil total nitrogen (∆ STN) with its canopy size across 5 sampling sites in drylands of Tibetan Plateau.
